# Lack of Diversity in Research on Females with Ehlers-Danlos Syndromes: Recruitment Protocol for a Quantitative Online Survey

**DOI:** 10.2196/53646

**Published:** 2024-05-02

**Authors:** Jennifer E Glayzer, Bethany C Bray, William H Kobak, Alana D Steffen, Judith M Schlaeger

**Affiliations:** 1 Department of Human Development Nursing Science College of Nursing University of Illinois Chicago Chicago, IL United States; 2 Institute for Health Research and Policy University of Illinois Chicago Chicago, IL United States; 3 Department of Obstetrics and Gynecology College of Medicine University of Illinois Chicago Chicago, IL United States

**Keywords:** Ehlers-Danlos syndrome, hypermobility, social media, recruitment, Facebook, hereditary disease, connective tissue disorders, racial, ethnic, diversity, challenges, strategies, strategy, online, information seeking, cross-sectional survey, dyspareunia, painful sex, United States

## Abstract

**Background:**

Ehlers-Danlos syndromes (EDS) are a group of connective tissue disorders caused by fragile lax collagen. Current EDS research lacks racial and ethnic diversity. The lack of diversity may be associated with the complexities of conducting a large international study on an underdiagnosed condition and a lack of EDS health care providers who diagnose and conduct research outside of the United States and Europe. Social media may be the key to recruiting a large diverse EDS sample. However, studies that have used social media to recruit have not been able to recruit diverse samples.

**Objective:**

This study aims to discuss challenges, strategies, outcomes, and lessons learned from using social media to recruit a large sample of females with EDS.

**Methods:**

Recruitment on social media for a cross-sectional survey examining dyspareunia (painful sexual intercourse) in females was examined. Inclusion criteria were (1) older than 18 years of age, (2) assigned female at birth, and (3) diagnosed with EDS. Recruitment took place on Facebook and Twitter (now X), from June 1 to June 25, 2019.

**Results:**

A total of 1178 females with EDS were recruited from Facebook (n=1174) and X (n=4). On Facebook, participants were recruited via support groups. A total of 166 EDS support groups were identified, 104 permitted the principal investigator to join, 90 approved posting, and the survey was posted in 54 groups. Among them, 30 of the support groups posted in were globally focused and not tied to any specific country or region, 21 were for people in the United States, and 3 were for people outside of the United States. Recruitment materials were posted on X with the hashtag #EDS. A total of 1599 people accessed the survey and 1178 people were eligible and consented. The average age of participants was 38.6 (SD 11.7) years. Participants were predominantly White (n=1063, 93%) and non-Hispanic (n=1046, 92%). Participants were recruited from 29 countries, with 900 (79%) from the United States and 124 (11%) from Great Britain.

**Conclusions:**

Our recruitment method was successful at recruiting a large sample. The sample was predominantly White and from North America and Europe. More research needs to be conducted on how to recruit a diverse sample. Areas to investigate may include connecting with more support groups from outside the United States and Europe, researching which platforms are popular in different countries, and translating study materials into different languages. A larger obstacle to recruiting diverse samples may be the lack of health care providers that diagnose EDS outside the United States and Europe, making the pool of potential participants small. There needs to be more health care providers that diagnose and treat EDS in countries that are predominantly made up of people of color as well as research that specifically focuses on these populations.

**International Registered Report Identifier (IRRID):**

RR1-10.2196/53646

## Introduction

### Overview

Ehlers-Danlos syndromes (EDS) are a group of hereditary connective tissue disorders [1]. The overall prevalence and racial and ethnic breakdowns of EDS are unknown [2]. It is estimated that EDS affects between 1 in 5000 [3] and 1 in 3400 [4] and is thought to be underdiagnosed instead of rare [4,5]. Females are diagnosed at a much higher rate than males with 7 females diagnosed for every 3 males [4]. It is unknown if this is due to the difference in presentation between the 2 sexes or how the condition is passed to offspring [6]. The inability to accurately determine the prevalence of EDS makes it difficult for investigators to recruit large diverse samples for studies, limiting the generalizability of results to participants whose demographics match study samples.

Most EDS studies have been comprised of predominately White samples recruited from the United States and Europe. Recruitment has traditionally been done at clinics or hospitals that treat patients with EDS [7], conferences [8-10], local in-person EDS support groups [11], or big data aggregation of national health records [4,12,13]. These recruitment methods limit the diversity of participants. Recruiting via clinics may limit participation to those who are physically and financially able to travel to the clinics; however, access to clinics is improving with the use of telemedicine. Some studies conducted via hospitals or clinics fail to report race or ethnicity demographics in their results [14-18]. Although national health record studies recruit from a large geographic area, to date they have only been completed in small northern European countries with predominantly White populations [4,12,13]. Extracting data from national health records also limits the types of data that can be analyzed to demographics and health care informatics, lacking metrics not included in patients’ charts such as symptom burden and personal experiences. Recruiting via EDS conferences includes only participants who are financially able and healthy enough to travel, excluding those who are of low socioeconomic status and the most ill. Furthermore, health care providers who treat and diagnose EDS and EDS researchers are predominantly located in the United States and Europe [19], which likely decreases the number of people diagnosed outside of these regions and, therefore, the number of people who would be eligible to participate in the research. The Ehlers-Danlos Society is an international nonprofit organization that is involved in raising awareness of EDS as well as supporting EDS research through research grants, patient education conferences, and health care provider symposia. The Ehlers-Danlos Society is based in the United Kingdom and the United States and operates in English, which may be a barrier for non-English speaking health care providers and researchers to participate in research and attend symposia. The Ehlers-Danlos Society is based in the United Kingdom and the United States and operates in English, which may be a barrier for health care providers and researchers in non–English-speaking countries for participating in research and attending symposia. However, the Ehlers-Danlos Society is actively trying to connect with or develop a network of EDS providers and researchers in countries where there are few EDS providers such as Japan [20]. Using social media for recruitment may facilitate the recruitment of diverse samples, increasing the generalizability of results to the global EDS population. However, studies conducted since this study in 2019 had similar results with most participants residing in the United States, Canada, the United Kingdom, and Australia [21-23].

### Use of Social Media for EDS Study Recruitment

EDS research has started to use social media for recruitment, but no literature has discussed research methods associated with this approach. A benefit of using social media to recruit is that it can reach a global population in a cost-effective manner [24]. Unable to find care for their symptoms and dismissed by health care providers [25,26], people with EDS often turn to support groups on social media to help them find physicians who diagnose and treat EDS, obtain tips about how to manage symptoms, and feel a sense of belonging [26]. The presence of online EDS support groups allows investigators to access groups of people with EDS. Without these support groups, people with EDS would have been difficult to reach due to the relatively low diagnosis rate. Social media lends itself well to recruiting for sensitive topics, such as medical conditions, because it provides a level of anonymity where individuals may be more comfortable disclosing information than if they had provided information in person [24,27].

### Purpose

The purpose of this study is to discuss the challenges, strategies, and outcomes of recruiting people with EDS on social media. We will discuss the protocol used to recruit participants for an online survey examining dyspareunia, pain during sexual intercourse, in people with vaginas who have EDS. The methods for this study in particular were chosen because of the successful recruitment of 1178 participants in 4 weeks, which enabled the research team to identify that vulvodynia occurs in half of females with EDS [28]. For this paper, the term EDS will be used to describe people with hypermobile EDS, hypermobility spectrum disorders, as well as other types of EDS. Not all people who have vaginas identify as female and the use of the terms “female” or “women” to describe all people with vaginas is inaccurate. However, for lack of a better term at this time, we will use the term female to describe people in this study.

## Methods

### Recruitment

We recruited for an open online survey examining dyspareunia in females who had EDS from June 1 to July 7, 2019. The survey, participant screening, and consent were completed on Qualtrics (Qualtrics). Details of the survey design and findings are reported elsewhere [28]. Inclusion criteria were (1) a self-reported diagnosis of EDS or a hypermobility spectrum disorder previously confirmed by a health care provider, (2) assigned to the female sex at birth and not had genital gender reassignment surgery, (3) 18 years of age or older, and (4) able to read English. The IP addresses were monitored to prevent people from participating more than once.

### Ethical Considerations

The study was approved by the University of Illinois Chicago institutional review board (IRB; #2019-0219). Informed consent took place on Qualtrics and involved participants reading a consent form and then selecting whether they agreed and wanted to participate or not. The study data collected were anonymous, and no protected health information or personal identifiers were collected. No compensation was provided for participation.

### Social Media Platform Choice

There are 3 basic types of social media platforms—text-based, photo or video-based, and private messaging. The social media quick guide (Table 1) outlines the basic functions and features of the most common social media platforms. Text-based platform posts are centered around text, but images and links can be attached. Image-based platform posts are centered around sharing photos or videos that can include text, but the photo or video is the main part of the post. Private messaging platforms are used to communicate privately between users and cannot be used to post information publicly. Text-based platforms such as Facebook (Meta Platforms) and Twitter (now X; Twitter Inc) facilitate discussion. Facebook works especially well for support groups because of the ability to make groups “private.” In private groups, members need to be approved by group administrators to join, group content can only be seen by group members, group content is not searchable via search engines such as Google (Alphabet Inc), and members can be banned for breaking rules. Private groups allow members to discuss sensitive topics with some privacy. X uses hashtags to organize public and private posts. Private posts can only be seen by a user's “friends,” people they have identified as knowing. X does not have a group function but instead, information can be searched via hashtags (#). For example, if a user searched for #puppies, all posts that included #puppies in the caption would appear. If a post is private, it would only appear in search results for people who have been identified as friends. Keeping posts private is helpful for discussing sensitive topics but eliminates discussion among members who are not “friends.”

**Figure 1:**
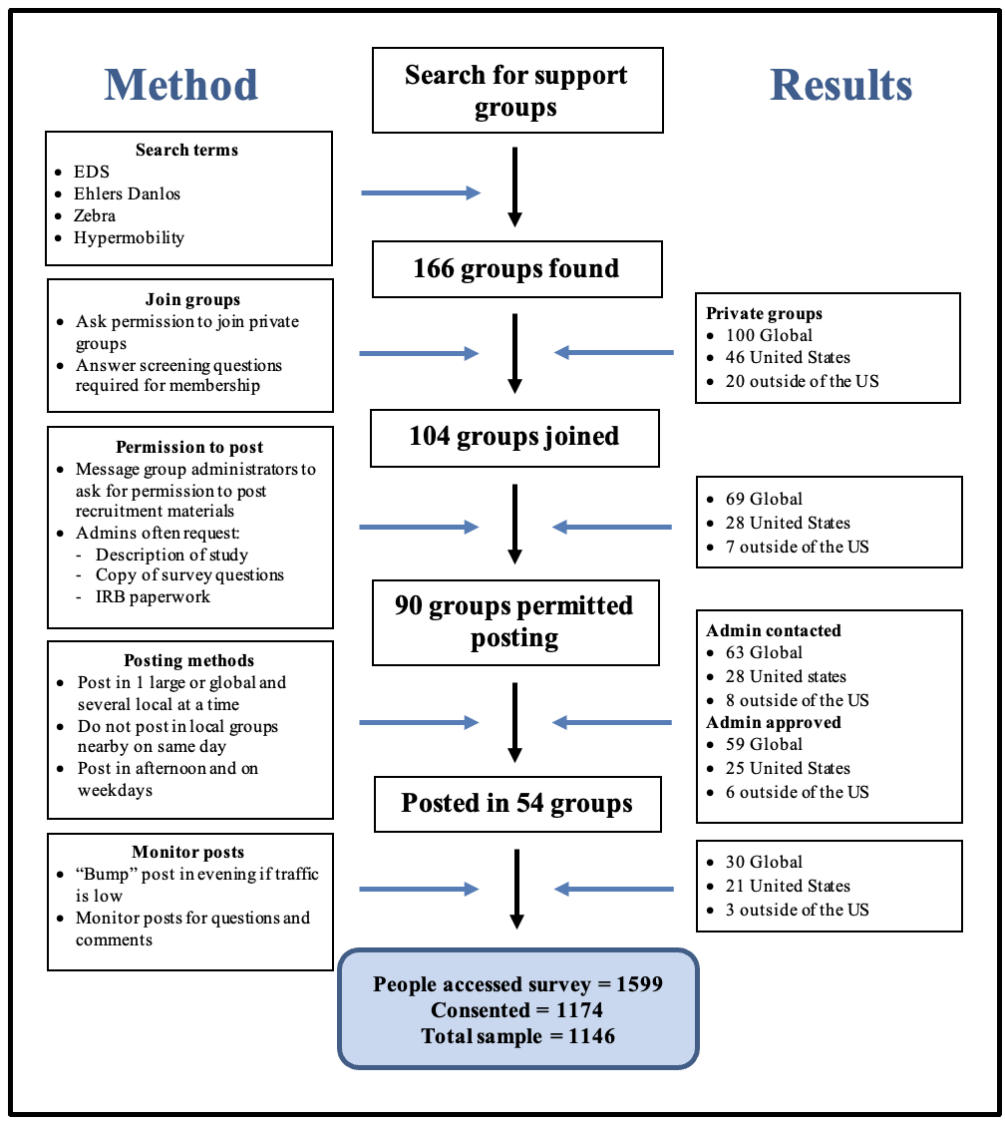
Facebook recruitment protocol and results. EDS: Ehlers-Danlos syndrome; IRB: institutional review board.

Facebook and X were selected for recruitment. Facebook was chosen because of the large number of existing EDS support groups. X was used to explore recruitment on social media via a platform that does not allow group formation. Facebook recruitment protocol and results (Figure 1) outline the recruitment protocol and results used for Facebook.

**Table 1:** Social media platform quick guide and features of common social media platforms.

Features				Facebook		Reddit		X		Instagram		TikTok		Snapchat		WhatsApp		WeChat
**Primary purpose**																		
	Text posts		✓		✓		✓											
	Image posts								✓									
	Video posts										✓							
	Private messaging												✓		✓		✓	
**Available features**																		
		Private posts	✓				✓		✓		✓							
		Group formation	✓		✓													
		Private group posts	✓															
		Posts searchable by topic or hashtags	✓		✓		✓		✓		✓							

### Searching for EDS Support Groups in Facebook

Search terms “Ehlers Danlos,” “EDS,” “Hypermobility,” and “Zebra” were used to search for EDS support groups on Facebook. Zebra was used as a search term because it is the mascot for EDS and other rare diseases. Zebra comes from the phrase taught to medical students that if you hear hoof beats think horse and not zebra, as it is more likely to be a horse. When this concept is applied to patients it can cause doctors to miss rare and underdiagnosed diseases such as EDS [29].

### Joining EDS Support Groups on Facebook

All support groups the primary investigator (PI) found were private. The PI requested permission to join the private groups. Private Facebook support groups frequently have users answer questions before granting or denying them membership. Groups often require people to have or know someone who has EDS to join. When requesting to join these groups, the PI disclosed that she was a researcher and would like to join the group for the purpose of recruiting for the study. Once the PI joined a group, the administrators of the group were messaged to ask for permission to post the recruitment materials. Messages included information about the PI and the project, and that the project was approved by an IRB. Although groups did not mandate permission before allowing posting, this approach provided transparency about the PI’s motives and helped to build trust between the group and the PI.

### Posting Recruitment Material on Facebook

Strategies developed based on previous Facebook use were used to try to reach the most participants possible. Recruitment materials were posted in 3 to 4 groups per day, generally 1 large group of over 1000 members and 3 small groups each with less than 1000 members. Groups in the same geographical area, for example, a Michigan support group and a Great Lakes support group were not posted in on the same day due to the likelihood of membership overlap. Posting recruitment materials in all groups that consented at the same time would limit the visibility of the post to people who were on social media at that time and cause users who are in several groups to see the same post in their feed multiple times and potentially cause them to become annoyed. A feed is like a home page that displays posts from a user’s friends and groups of which they are a part. Posts are displayed in chronological order with the newest and most active posts appearing at the top. Active posts are posts that have been commented on or liked. You may comment on your own post to help it move up in a feed; this is called bumping. Posting in different groups over several days will increase the time a post is at the top of a feed for users who are in more than 1 group.

### Posting Recruitment Material on X

On X, hashtags related to EDS were searched. Recruitment materials were posted with #EDS by an account specifically made for the study. X posts are limited to 280 characters, requiring a shorter study description than Facebook posts. Prior to posting, the PI interacted with and followed other X accounts related to EDS and pain. X accounts were found by searching for accounts that included search terms in their profile or were included in post hashtags. The same search terms used to search for Facebook support groups were used here. This increased the account’s presence and boosted the number of users who would see the posts.

### Monitoring Recruitment Posts

All posts on Facebook and X were monitored and any questions or comments were addressed quickly. Responding quickly could keep the post at the top of a user’s or group’s feed and hopefully address the user’s question before they left their social media account and lost interest in participating. Once recruitment goals were met, the PI stopped contacting groups for permission to post and stopped posting recruitment materials.

### Statistical Analysis

Recruitment data were analyzed from 3 sources—Facebook search results, Facebook group tracking, and survey response data. Excel (Microsoft Corp) was used to track (1) the name of each group, (2) the size of each group, (3) if permission was given to join the group, (4) who was contacted to ask for permission to post in the group, (5) when the person was contacted, (6) if permission was given to post in the group, and (7) when recruitment materials were posted in the group. Stata Software for Statistics (version 15; StataCorp) was used to analyze descriptive statistics of the search results from the Facebook EDS support groups search. Survey response data were exported from Qualtrics to Excel to be cleaned and then to Stata 15 for analysis. For the original study examining dyspareunia, participants missing key data were not included. For this study, all participants who consented were included. Survey response data were used to analyze participant demographics and their traffic. The traffic of participants was analyzed by calculating the number of participants who accessed the survey during the 6-hour time blocks of 4 AM to 9:59 AM, 10 AM to 3:59 PM, 4 PM to 9:59 PM, and 10 PM to 3:39 AM EST.

## Results

### Facebook Recruitment

The Facebook recruitment protocol and results (Figure 1) provide the number of groups included in each step of the recruitment process. The EDS support groups search yielded 166 EDS Facebook support groups ranging in size from less than 100 to 33,000. Of the 166 groups found, 104 (63%) gave permission for the PI to join. Common questions asked by group administrators when the PI requested permission to join groups included the following: *Why do you want to join the group?*, *Do you or someone you know have EDS?*, *Do you live in XX city (if the group was focused on a specific geographic area)?*, and *Do you agree to follow the rules of the group?* Group rules often included not taking screenshots of group conversations, not disclosing the identity of members to nonmembers, and no soliciting.

Of the 104 groups that gave permission, 90 (86%) gave permission to post the recruitment materials. Three of the groups that did not permit the PI to join offered to post recruitment materials for the PI. The PI was denied access to post in several groups for cisgendered language in the survey and recruitment materials. These groups preferred the use of specific organs instead of the terms “woman” and “female.” Of the 90 groups that gave permission to post, 54 (60%) were posted in before recruitment goals were met. Once the recruitment goals were met posting was stopped by the PI.

When analyzing the data, EDS support groups were divided into 3 categories—global (or no specific geographic location specified); United States for users who resided in the United States (eg, Chicago EDS support group); and outside of the United States for users who resided outside of the United States (eg, Sussex, England EDS support group). The categories of United States and outside of the United States were used because of the high prevalence of US groups and the sparseness of groups from other specific countries. Global groups were separated from the US groups and groups outside of the United States because global groups have different focuses than region-specific groups. Groups specific to a geographic location can be used to help members find local health care providers and members are able to meet in person. Global groups are often focused on broad topics such as comorbid conditions associated with EDS. Global groups were larger in size and more prevalent than the other 2 categories, with 100 global groups found compared to 46 US groups and 20 outside of the US groups. Of the 54 groups in which recruitment materials were posted, 30 (56%) were global groups, 21 (39%) were US groups, and 3 (18%) were outside of the US groups.

### User Statistics

**Figure 2:**
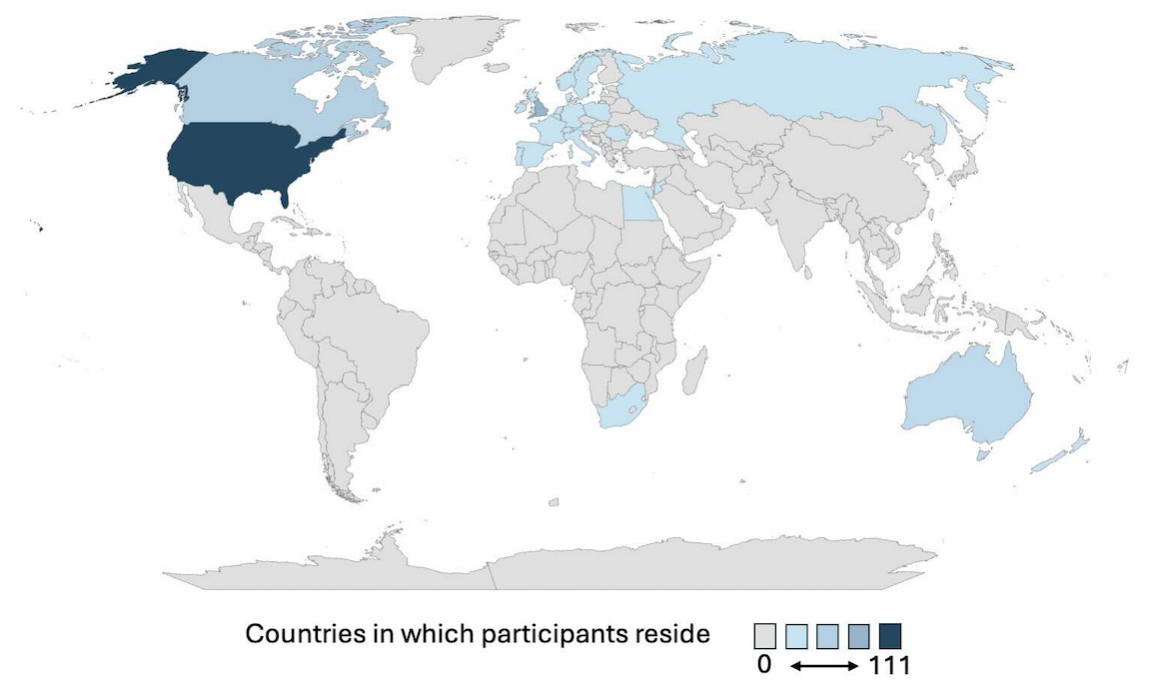
Heat map of countries in which participants reside.

There was a total of 110,665 users in the 54 groups posted in; however, this does not take into consideration that users are often members of more than 1 group. The smallest group posted in had 47 members and the largest had 33,170 members. We were unable to calculate a response rate because we were unable to determine how many users saw each post and then accessed the survey. Of the 110,665 people who potentially saw the post, 1599 accessed the survey and 1178 females were eligible, consented, and included in analyses. Of the 1178 consented participants, 1174 were recruited from Facebook and 4 from X. The average age of participants was 38.6 (SD 11.7) years, similar to the average age of participants in big data studies of EDS [4,12,13,30]. Participants were predominantly White (n=1063, 93%), non-Hispanic (n=1046, 92%), and had the most common form of EDS, hypermobile EDS (n=1290,79%) [2]. Participants were recruited from 29 countries with 900 (76%) from the United States and 124 (11%) from Great Britain. The map of the participant’s country of residence (Figure 2) shows all the countries from which the participants were recruited. Participant characteristics can be found in the characteristics of the sample (Table 2) [28].

**Table 2:** Characteristics of the sample (number of females with Ehlers-Danlos syndromes; N=1178) [17].

Demographics		Values
**Age (years; n=1141)**		
	Mean (SD)	38.6 (11.7)
	Range	18-76
**Sex, n (%)**		
	Female	1144 (99.8)
	Transgender	2 (0.2)
**Race^a^, n (%)**		
	White	1063 (92.8)
	Black or African American	12 (1.0)
	American Indian or Alaskan Native	17 (1.5)
	Asian	11 (1.0)
	Native Hawaiian or Pacific Islander	3 (0.3)
	Other	47 (4.1)
**Ethnicity (n=1138), n (%)**		
	Hispanic or Latino	41 (3.6)
	Not Hispanic or Latino	1046 (91.9)
	Unknown or not reported	51 (4.5)
**Country of residence, n (%)**		
	Unites States	900 (78.5)
	Great Britain	124 (10.8)
	Canada	46 (4.0)
	Australia	23 (2.0)
	Other	66 (5.7)

^a^Participants selected all races they identified with.

### Survey Traffic

To examine when participants from different parts of the world accessed the survey and determine optimal posting times for recruiting participants globally, we analyzed what time participants accessed the survey. What time participant accessed the survey was analyzed by time of day and by day of week for the whole sample (N=1178), those residing in North America (n=971), and those residing outside of North America (n=206). The PI posted recruitment materials at different times during the day and night in hopes of recruiting people in different time zones. The North America and non–North America groupings were used for analysis instead of the country of residence due to data sparseness and overlapping time zones of the countries. The busiest time block for recruitment of participants as a whole (n=465, 39%) and among participants in North America (n=411, 42%) was 4 PM to 9:59 PM. Recruitment was busiest from 10 AM to 3:59 PM for participants outside of North America (n=57, 17%). Recruitment of participants outside of North America was more evenly distributed across all time blocks with about 50 participants per time block. The 4 AM to 9:59 AM time block had the least amount of traffic for the whole group (n=130, 11%), participants from North America (n=87, 9%), and participants from outside of North America (n=43, 4%). Monday was the busiest recruitment day for all groups with 301 (25%) participants from the whole sample, 244 (25%) from North America, and 57 (27%) from outside of North America. Saturday through Tuesday were busier than Wednesday through Friday. The day of the week that participants from North America were recruited differed significantly compared to participants from outside of North America (*χ*^2^_6_=34.1; *P≤*.001).

## Discussion

### Principal Findings

We were able to recruit a large sample in a short amount of time, highlighting the usefulness of using social media, specifically Facebook EDS support groups and X, for recruitment. Our method of recruiting via Facebook can be used to target populations that have access to the internet and use social media. Support groups on social media provide curated groups of people that would otherwise be difficult to find, facilitating recruitment of large groups of participants for rare and underdiagnosed conditions. However, like other EDS studies that recruited via social media, we were not successful at recruiting a diverse sample with our participants being predominantly from the United States and White. Our results do provide greater detail about the countries participants resided in when they accessed the survey, and their races and ethnicities. Many of the large studies conducted in Northern Europe do not provide demographic details such as race and ethnicity. Providing demographic information is key to assessing the diversity of a sample and evaluate generalizability. Demographic information including race and ethnicity should be included in every study’s results. Future directions for research to increase diversity in a sample may include investigators connecting with more support groups from outside of the United States and Europe, use of social media platforms that are popular in other countries, and translating study materials into different languages. More fundamental obstacles potentially include the need for more health care providers that diagnose and treat EDS in countries that are predominantly comprised of people of color, and the need to conduct research that targets these populations.

### Challenges That Were Successfully Addressed

Some social media platforms are better than others for recruiting participants. Facebook worked well for recruiting because existing EDS support groups had thousands of members. Posting in these groups allowed the PI to reach many people with EDS with each post. When posting in groups on Facebook, it is easy to see how active a group is based on how frequently people are posting in the group and how many comments and likes the posts receive. Posting in groups on Facebook was chosen over platforms where hashtags (#) are used because these platforms do not have existing groups of people with EDS. On platforms where hashtags are used, the visibility of a post for users is influenced by the number of followers users have. Therefore, for a researcher to reach a lot of people with a post they must create a profile, as well as cultivate a following which can take a fair amount of time. It is also difficult to determine which hashtags are most popular and how people are interacting with hashtags. A better way to share a study on platforms that use hashtags, such as TikTok (ByteDance), is to have existing platform users, with a large group of followers, share the study on their social media page, such as EDS nonprofits.

Our recruitment protocol helped to efficiently keep track of where we were in the recruitment process and within each support group. Documenting the name of each group administrator and when they were contacted allowed the PI to quickly determine what chat conversation was associated with each support group because chats are labeled with users’ names and not their affiliated support group. It was also difficult to decide what and when support groups should be posted in. Having the size, where each group was located, and if the group had a specific focus helped determine what groups to post in on a given day. Asking for permission to post recruitment materials in groups was the most labor-intensive aspect of recruitment. On Facebook, messages from users who are not your “friend” are sent to a message request folder that is not easily seen. Typically, when using Facebook Messenger, conversations with “friends” are kept in 1 location and users receive a notification when they receive a new message. This does not happen when a user receives a message from someone who is not a friend. When a message is seen by a recipient in Facebook Messenger, their profile picture appears next to the message. To address the challenge of messages going unseen by group administrators, the PI contacted a different group administrator. Administrators of smaller local groups tended to respond to messages more often and more quickly.

### Challenges That Still Need to Be Addressed

#### Recruiting Participants From Diverse Countries of Residence

Efforts to recruit participants quickly may have led to decisions that decreased the diversity of participants. A total of 21 (45%) of the EDS support groups posted in were US support groups. Anecdotally it seemed easier to recruit from small local EDS support groups because they were more active than global groups, determined by users’ engagement such as commenting and liking posts. Support groups that were focused on areas outside of the United States were stricter about allowing people from outside their area to join and post, with 6 (30%) of groups outside of the United States permitting posting compared to 59 (59%) of global groups and 25 (54%) of US groups. Establishing relationships with EDS communities in other countries, to gain permission for research recruitment, may lengthen recruitment. Whether or not establishing these relationships would increase diversity in a sample needs to be assessed. A limitation is that the PI only posted in 3 (50%) of the groups outside of the United States that gave permission before stopping recruiting due to meeting recruitment goals. The decision to post in local US groups over groups based in other countries was based in part on the perception that groups outside of the United States received less traffic. Instead of using groups located outside of the United States, due to perceived low activity, larger more active global groups were targeted. However, it is important to consider that even though a group is global, it may have a large percentage of users from the United States. In the future, global groups and groups outside of the United States should both be a priority for recruiting. It may be beneficial to work with local collaborators, researchers, or advocates who are familiar with the local support groups in their respective countries. Furthermore, social media preferences may differ between countries.

#### Racial and Ethnic Diversity Within the Sample

Definitions of race and ethnicity vary across the world. This study used the race and ethnicity categories from the US National Institutes of Health with an “other” option added. Identifying racial categories that are used globally and less focused on the United States may make it easier to compare demographics from studies conducted in other countries and may be more inclusive. Our sample was predominately White (n=1063, 93%) The racial and ethnic breakdown of EDS is unknown [2]. In a study looking at EDS diagnoses in hospitalized patients in the United States, 1736 of 2007 (86%) participants were White [30]. However, in the United States, race and ethnicity affect access to care, including receiving an EDS diagnosis and having an inpatient hospital admission, which may cause an overestimation of the percentage of people with EDS who are White. Similarly, there is decreased access to the internet in rural and low-income areas in the United States and abroad. This decreased access disproportionately affects minorities within the United States and people of color abroad, limiting their participation [31,32]. Big data studies using national health records in predominantly White northern European countries did not report race or ethnicity demographics [4,12,13]. Our high percentage of White participants was likely driven by recruitment from the United States, where structural inequities affect access to care, as well as northern European countries that have predominantly White populations. The lack of access to care and doctors who diagnose EDS outside of North America and Europe [19] likely limits the pool of people from these areas who meet the inclusion criteria of an EDS diagnosis, and therefore, may have impacted the diversity of our study. Similarly, due to the changing terminology for hypermobile EDS and hypermobility spectrum disorders, it is important to include participants with both diagnoses, as a participant’s diagnosis may vary based on when or where they were diagnosed, as well as what type of health care provider diagnosed them. There needs to be more health care providers that diagnose and treat EDS in countries that are predominately made up of people of color along with research focused on these specific populations. Research in these groups should be facilitated by members of the EDS community from these groups.

#### Including LGBTQIA+ Communities

Specific EDS support groups for lesbian, gay, bisexual, transgender, queer or questioning, intersex, asexual, people, as well as those who not listed but identify as part of this community, were targeted for recruitment; however, we were not permitted to post in a few groups because of cisgendered language in our recruitment materials and survey. Facebook EDS support groups preferred the use of gender-neutral language based on the specific organs needed to participate rather than the terms “woman” and “female.” To ensure the study is in line with the EDS communities’ values and inclusive of nonbinary individuals, researchers should include community representatives in the development of their study.

### Limitations

As with other EDS research, our sample was predominately White and from the United States. We were limited by the lack of support groups in countries that were not predominately White. In future research, non-US support groups should receive more focus than US-specific groups. The use of social media platforms likely varies between countries. A wider variety of social media platforms should be explored for use.

The use of social media platforms likely varies between countries. A wider variety of social media platforms should be explored in future research. Additionally, participants were recruited for this survey in 2019, since then there have been many changes in social media including Twitter changing to X and the rise of TikTok. However, the lack of diversity in EDS research persists even as recruitment on social media increases. Identifying recruiting obstacles can help develop methods to overcome them for more diverse and inclusive research.

The survey was conducted without verification of participants’ EDS diagnosis and, therefore, may have included people who have not been diagnosed with EDS. For more rigorous and valid results, a participant’s EDS diagnosis should be verified; however, this may be difficult via social media. Recruiting via social media may work best for studies that are exploring new areas of research and identifying new phenomena that can then be followed up with more rigorous methods.

During recruitment, an IRB amendment was needed. Until the amendment was approved, recruitment was paused. When recruitment was paused, prospective participants were still able to see recruitment posts, and this caused some confusion. To mitigate confusion, we edited the posts to explain recruitment was temporarily paused. We posted again when recruitment resumed. Commenting on the post that recruitment was live again should have brought the post back up to the top of feeds.

### Conclusions

Social media is effective and efficient at recruiting people with EDS for research studies. Text-based platforms that have established EDS support groups such as Facebook are well-suited for recruiting. Specific attention should be paid to recruiting people outside of the United States. Considerations for research on recruitment of participants with EDS include (1) working with local researchers and communities where you wish to recruit from, (2) posting during peak social media traffic time in target areas, and (3) increasing diagnoses and care of patients, which will increase awareness of EDS outside of the United States and Europe. Working with researchers, clinicians, and advocates in areas with support groups outside of the United States may help researchers gain permission to post in groups in these regions and provide guidance on where best to recruit people in their area, as well as ensure survey materials are in line with the EDS community’s values. Posting during peak traffic for people outside of North America may help recruit a more global sample. There is still little research on people with EDS outside of the United States and Europe. To engage participants outside of these regions, research materials may need to be translated into other languages. A repository of tools used to evaluate written materials related to EDS in many languages would make it easier for researchers to recruit participants who speak different languages, as well as make results easier to compare across studies. A larger issue may be a lack of EDS providers and researchers in countries outside of the United States and Europe. Research needs to be conducted examining the prevalence of EDS in these areas along with increasing awareness of the condition. Recruiting people with EDS in countries where there are few if any health care providers who diagnose and treat EDS will remain a challenge until awareness of EDS grows and care improves.

## Abbreviations

EDS: Ehlers-Danlos syndrome
IRB: institutional review board
PI: primary investigator

## Data Availability

The data sets generated and analyzed during this study are available from the corresponding author upon reasonable request.
